# VLA-4 Induces Chemoresistance of T Cell Acute Lymphoblastic Leukemia Cells via PYK2-Mediated Drug Efflux

**DOI:** 10.3390/cancers13143512

**Published:** 2021-07-14

**Authors:** Sofiane Berrazouane, Alexie Doucet, Marc Boisvert, Frédéric Barabé, Fawzi Aoudjit

**Affiliations:** 1 Division of Immune and Infectious Diseases, CHU de Québec-Université Laval Research Center, Québec City, QC G1V 4G2, Canada; Sofiane.Berrazouane@crchudequebec.ulaval.ca (S.B.); alexie.doucet@crchudequebec.ulaval.ca (A.D.); marc.boisvert@cervo.ulaval.ca (M.B.); frederic.barabe@crchudequebec.ulaval.ca (F.B.); 2 Department of Medicine, Faculty of Medicine, Université Laval, Québec City, QC G1V 0A6, Canada; 3 Department of Microbiology-Infectiology and Immunology, Faculty of Medicine, Université Laval, Québec City, QC G1V 0A6, Canada

**Keywords:** VLA-4, VCAM-1, chemoresistance, T-cell acute lymphoblastic leukemia, PYK2, drug efflux

## Abstract

**Simple Summary:**

Cellular adhesion plays an important role in the development of resistance to chemotherapy (chemoresistance) that represents a major hurdle in the treatment of leukemia and which is a major cause for patient relapse. In this study, we evaluated if cell adhesion to the molecule VCAM-1, which is present in the leukemia microenvironment, can favour the chemoresistance of T acute lymphoblastic leukemia (T-ALL). Our results showed that adhesion of T-ALL cells to VCAM-1 via their receptor VLA-4 induces the resistance of T-ALL cells to doxorubicin by activating the signaling protein PYK2 but not FAK. VLA-4/PYK2 signaling did so by inducing the efflux of doxorubicin. However, adhesion of T-ALL cells to fibronectin via the receptor VLA-5 did not activate PYK2 and had no effect on doxorubicin resistance. These findings suggest that targeting the VLA-4/PYK2 pathway could overcome T-ALL chemoresistance and reduce the risk of patient relapse.

**Abstract:**

Cell adhesion plays a critical role in the development of chemoresistance, which is a major issue in anti-cancer therapies. In this study, we have examined the role of the VLA-4 integrin, a major adhesion molecule of the immune system, in the chemoresistance of T-ALL cells. We found that attachment of Jurkat and HSB-2 T-ALL cells to VCAM-1, a VLA-4 ligand, inhibits doxorubicin-induced apoptosis. However, their adhesion to fibronectin, which is mainly mediated via VLA-5, had no effect. Even the presence of the chemoattractant SDF1α (Stromal cell-derived factor-1α), which enhances the adhesion of T-ALL cells to fibronectin, did not modify the sensitivity of the cells attached on fibronectin towards doxorubicin-induced apoptosis. Mechanistically, we found that VLA-4 promoted T-ALL chemoresistance by inducing doxorubicin efflux. Our results showed that cell adhesion to both fibronectin and VCAM-1-induced Focal adhesion kinase (FAK) phosphorylation in T-ALL cells. However, only cell adhesion to VCAM-1 led to PYK2 phosphorylation. Inhibition studies indicated that FAK is not involved in doxorubicin efflux and chemoresistance, whereas PYK2 inhibition abrogated both VLA-4-induced doxorubicin efflux and chemoresistance. Together, these results indicate that the VLA-4/PYK2 pathway could participate in T-ALL chemoresistance and its targeting could be beneficial to limit/avoid chemoresistance and patient relapse.

## 1. Introduction

Integrins are α/β heterodimeric transmembrane receptors that mediate cell–cell and cell–matrix adhesion. Upon ligand binding, integrins activate focal adhesion kinases including FAK and PYK2, leading to the activation of downstream pathways to regulate cell migration, survival and proliferation [1,2,3,4]. 

VLA-4 or α4β1 integrin is a major integrin in the immune system. It plays a critical role in lymphocyte development and activation, as well as in the trans-endothelial migration of leukocytes into inflammatory sites [5,6,7]. VLA-4 binds to two major ligands including vascular adhesion molecule 1 and fibronectin that can be expressed by various cells including antigen-presenting cells, endothelial and stromal cells [8,9,10].

In hematological malignancies, VLA-4 integrin has been reported to enhance the homing and dissemination of leukemic cells into the bone marrow and other peripheral sites [11,12]. It also mediates the adhesion of leukemic cells to bone marrow mesenchymal stem cells (BM-MSCs) [13,14]. This adhesive interaction enhances leukemic cell survival and proliferation, as well as their resistance to chemotherapy (chemoresistance). In this respect, VLA-4 integrin induces the chemoresistance of acute myeloid leukemia, myeloma and B cell malignancies [15] and is a strong predictor of patient relapse and poor prognosis in B cell chronic leukemia [16] and childhood B-cell precursor acute lymphoblastic leukemia [17]. VLA-4-mediated chemoresistance in these malignancies is associated with NF-κB (Nuclear factor kappa B) activation and upregulation of Bcl-2 (B-cell lymphoma 2) pro-survival proteins [18]. 

VLA-4 is also strongly expressed in T-acute lymphoblastic leukemia (T-ALL) [19]. Previous studies have shown that T-ALL cells interact with VCAM-1 via VLA-4, whereas they attach to fibronectin mainly through the fibronectin-binding integrin VLA-5 with no or little contribution of VLA-4 [20,21]. VLA-4 has been implicated in the survival of T-ALL blasts upon adhesion to BM-MSCs [22]. However, adhesion to VCAM-1 enhances activation-induced cell death of T cell clones [23]. On the other hand, and in contrast to collagen, which acted via VLA-2 integrin, fibronectin failed to protect Jurkat T cells from activation-induced apoptosis [24] and from chemotherapy [25]. Whether VLA-4 promotes the chemoresistance of T-ALL cells is still unclear.

In this study, we used VCAM-1 as well as fibronectin to discriminate VLA-4 from VLA-5 and evaluated the resistance of T-ALL cells to doxorubicin. We found that attachment of T-ALL cells to VCAM-1 protected them from doxorubicin-induced apoptosis by activating doxorubicin efflux in a PYK2-dependent manner. In contrast, attachment to fibronectin, which is dependent mainly on VLA-5, did not activate PYK2 and had no effect on the sensitivity of T-ALL cells. These results suggest that activation of the VLA-4/PYK2 pathway can contribute to the generation of drug resistance in T-ALL. 

## 2. Materials and Methods

### 2.1. Reagents and Antibodies

Recombinant human VCAM-1/CD106 Protein and Recombinant Human/Rhesus Macaque/Feline CXCL12/SDF1α were from R&D Systems (Minneapolis, MN, USA). Human fibronectin, doxorubicin and FAK inhibitor PF-228 were purchased from Millipore Sigma (Oakville, ON, Canada). The PYK2 inhibitor VS-6063 was purchased from Cayman Chemicals (Ann Arbor, MI, USA). Anti-phospho-PYK2 (Tyr-402) and anti-PYK2 antibodies were purchased from Cell Signaling Technology (Danvers, MA, USA). The fluorescein isothiocyanate (FITC)-conjugated Annexin V, Phycoerythrin (PE)-conjugated anti-human α4 integrin, Alexa 647-conjugated anti-human α5 integrin antibodies, FITC-conjugated anti-human CD3 and PE-conjugated anti-mouse IgG were purchased from BD Biosciences (San Jose, CA, USA). Anti-human β1 integrin (4B4) was purchased from Beckman Coulter Life Sciences (Mississauga, ON, Canada).

### 2.2. Cell Culture

The human T-ALL cell lines Jurkat (E6.1) and HSB-2 were purchased from ATCC (Manassas, VA, USA) and were cultured in RPMI 1640 medium with 10% of fetal bovine serum (FBS), 100 units/mL penicillin, streptomycin and 2 mmol/L glutamine (complete medium).

### 2.3. Determination of α4, α5 and β1 Integrin Expression 

Jurkat and HSB-2 cells were incubated for 45 min at 4 °C in PBS containing 1% FBS with FITC-conjugated anti-human CD3, PE-conjugated anti-human α4 integrin or with Alexa 647-conjugated anti-human α5 integrin antibodies. For β1 integrin staining, the cells were first incubated with anti-human β1 integrin (4B4) for 45 min, washed and then incubated with PE-conjugated anti-mouse IgG for 45 min. After incubation, the cells were washed twice with PBS and analyzed by flow cytometry using a FACSCalibur II cytometer (BD Biosciences).

### 2.4. Analysis of Apoptosis and Drug Efflux by Flow Cytometry

VCAM-1 (25 µg/mL), fibronectin or BSA (Bovine serum albumin) (500 µg/mL) diluted in PBS were coated in 96 well-plates overnight at 4 °C. The wells were washed and Jurkat and HSB-2 resuspended in RPMI medium without FBS at 2 × 10^6^ cells/mL were seeded and cultured for 4 h. In some wells, the cells were also activated with 200 ng/mL of SDF1α. Then, cells in suspension were removed by washing with RPMI medium and the adherent cells were treated with doxorubicin in complete RPMI medium at a final concentration of 50 ng/mL and 10 ng/mL for Jurkat and HSB-2 cells respectively. After 48 h, cell apoptosis was determined by FITC-conjugated Annexin V staining and analyzed by flow cytometry using a FACSCalibur cytometer (BD Biosciences).

### 2.5. Doxorubicin Efflux

To assess doxorubicin efflux, T-ALL cells were cultured on BSA, VCAM-1 or fibronectin for 4 h and were then treated for 2 h with 100 ng/mL of doxorubicin. The cells were recovered and washed twice with PBS. Doxorubicin is naturally fluorescent and the intracellular doxorubicin content is analyzed by the FACSCalibur cytometer by using the FL-2 channel. The data are presented as the percentage of positive cells times MFI (mean fluorescence intensity) as previously described [26].

### 2.6. Cell Adhesion Assays

Jurkat and HSB-2 cells were activated or not with SDF1α and cultured in wells coated with VCAM-1, fibronectin or BSA. After 4 h, the wells were washed three times with RPMI medium and the remaining adherent cells were detached and counted microscopically.

### 2.7. Activation of Focal Adhesion Kinases 

The phosphorylation of FAK (Y397) and Pyk2 (Y402) was determined by specific ELISA assays from RayBiotech (Peachtree Corners, GA, USA) according to the manufacturer’s instructions.

### 2.8. PYK2 Activation and Western Blot

Cells were cultured on BSA, VCAM-1 or fibronectin for 15 and 30 min, harvested and lysed with RIPA buffer supplemented with protease and phosphatase inhibitors. PYK2 activation was analyzed by immunoblot by utilizing the anti-phosphorylated-PYK2 (Tyr-402) antibody. The blots were then stripped and reprobed with anti-PYK2 antibody to ensure equal loading. The bands corresponding to phosphorylated PYK2 and total PYK2 were subjected to densitometry quantification with the Molecular Imager^®^ Gel Doc™ XR System from Bio-Rad Laboratories (Mississauga, ON, Canada). Uncropped western blots for Figure 7 can be found from Supplementary Figure S3.

### 2.9. Statistical Analysis

Statistical analysis was performed by the Student’s t-test and two-way ANOVA test. *p*-values < 0.05 were considered significant.

## 3. Results

### 3.1. Expression of VLA-4 (α4β1) and VLA-5 (α5β1) Integrins and Adhesion of Human T-ALL Cell Lines to VCAM-1 and Fibronectin

**Figure 1:**
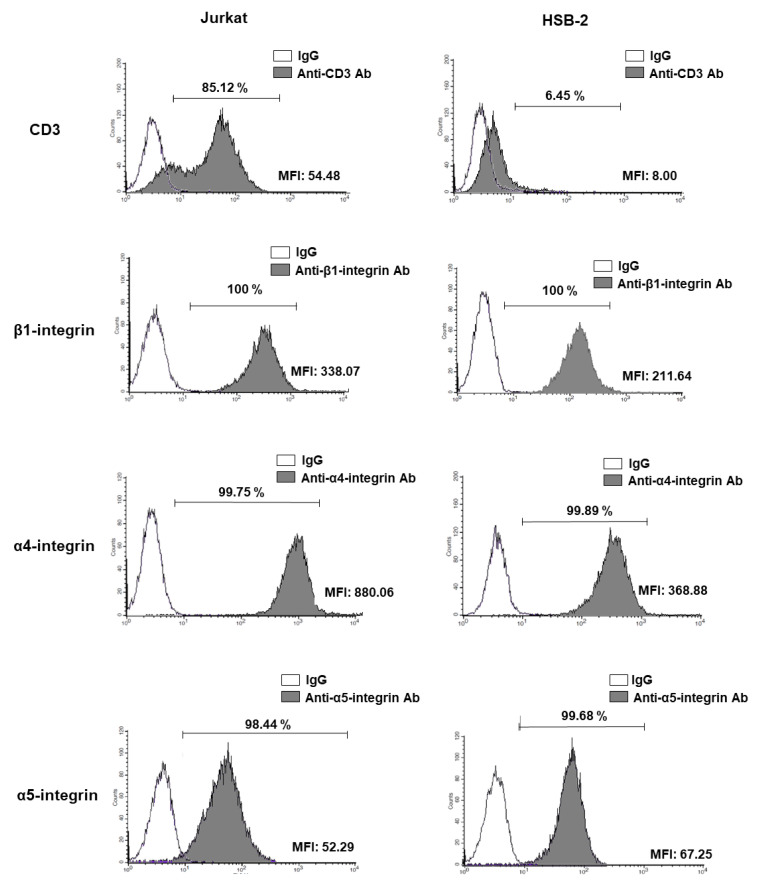
α4, α5 and β1 integrin chain expression in human T-ALL cell lines.

We first examined the expression of VLA-4 and VLA-5 in two well-described T-ALL cell lines Jurkat and HSB-2 with a mature and immature phenotype respectively. Consistent with previous observations, CD3 staining indicates that more than 85% of Jurkat cells are positive for CD3 compared to only 6.45% of HSB-2 cells correlating with the mature phenotype of Jurkat vs. HSB-2 cells (Figure 1). β1, α4 and α5 integrin chains are highly expressed in almost 100% of Jurkat and HSB-2 cells. However, Jurkat cells express higher levels of α4 integrin with a MFI: 880 compared to a MFI of 368 for HSB-2 cells (Figure 1). 

**Figure 2:**
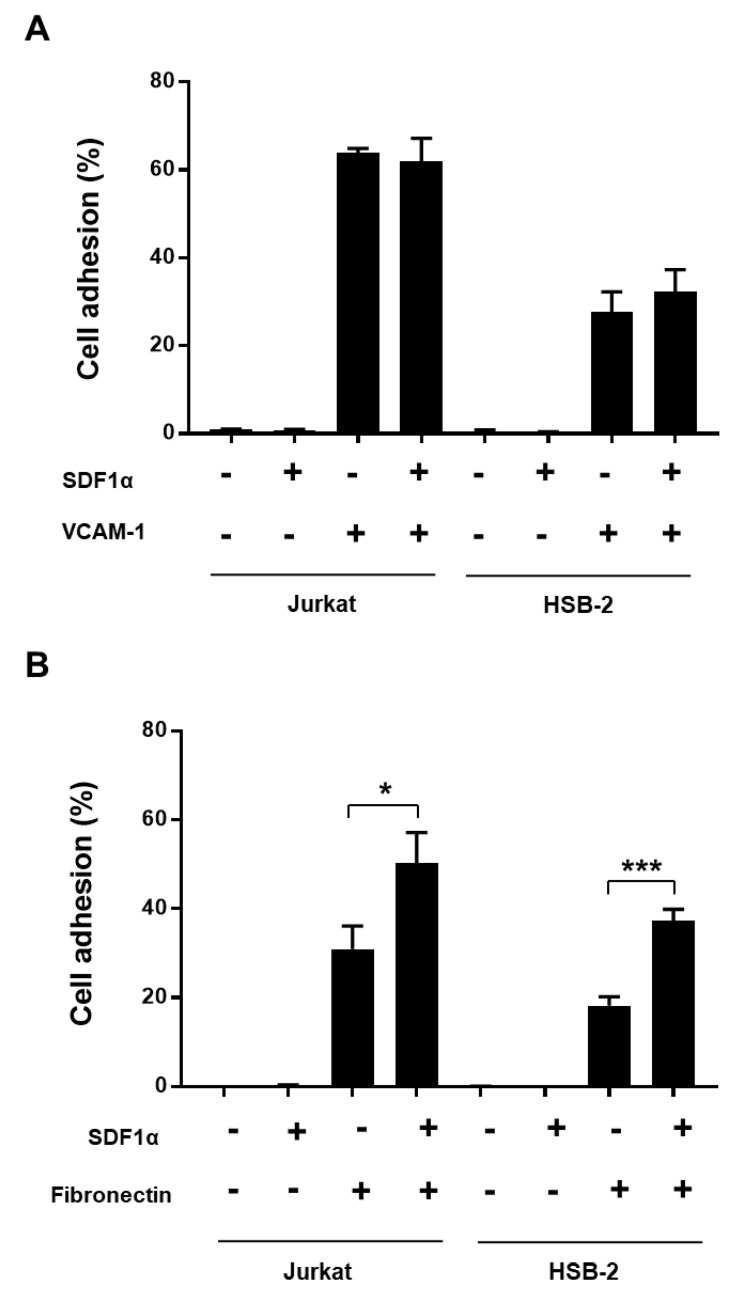
T-ALL cell adhesion to VCAM-1 and fibronectin. Jurkat and HSB-2 cells were pre-activated or not with SDF1α for 1 h and then seeded on wells coated with VCAM-1 (**A**), fibronectin (**B**) or BSA (−) for 4 h. The cells were washed three times and the remaining cells were counted. Results represent mean values percentages of adherent cells ± S.D. of three independent experiments. * *p* < 0.05 and *** *p* < 0.001 determined by the Student’s t-test.

We then assessed the percentage of Jurkat and HSB-2 cells adhering on VCAM-1 and fibronectin. The results show that the adhesion of Jurkat cells on VCAM-1 or on fibronectin is higher than that of HSB-2 cells (Figure 2). Almost 60% of Jurkat cells attach to VCAM-1 compared to only 30% of HSB-2 cells (Figure 2A). The presence of SDF1α, an important chemokine in T cell adhesion and migration and in T-ALL progression [27,28], had no effect on Jurkat and HSB-2 cell adhesion to VCAM-1 but increased by twofold the adhesion of both cells lines to fibronectin (Figure 2B). The use of functional blocking antibodies against α4 and α5 integrins confirmed previous observations [20,21] that T-ALL cell adhesion to VCAM-1 is mediated via VLA-4, whereas adhesion to fibronectin relies primarily on VLA-5 (Supplementary Figure S1).

### 3.2. VCAM-1 but Not Fibronectin Induces Resistance of T-ALL Cell Lines to Doxorubicin via Activation of Drug Efflux

**Figure 3:**
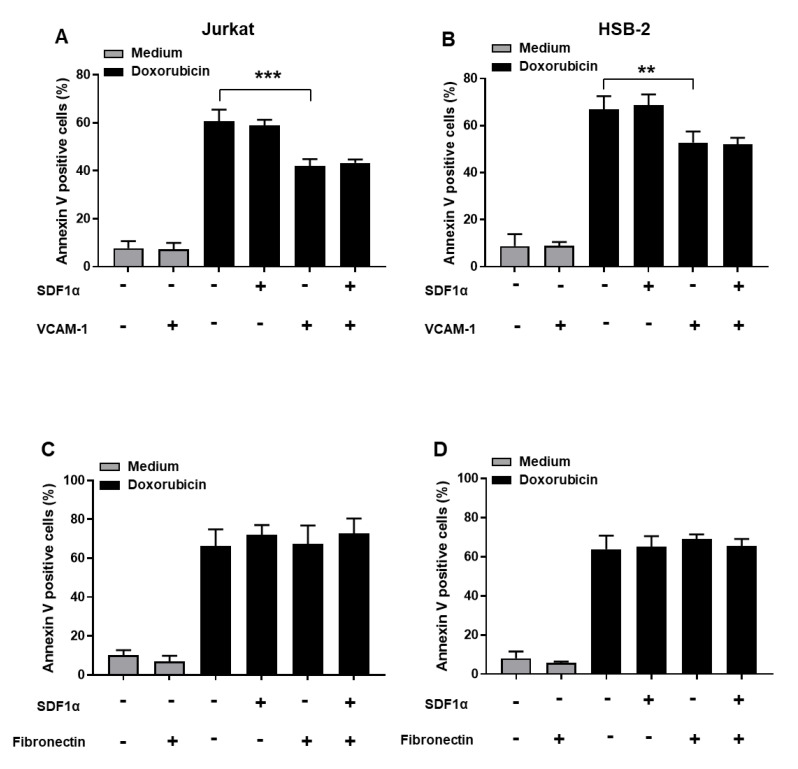
VCAM-1 but not fibronectin induces resistance of human T-ALL cell lines to doxorubicin. Jurkat (**A**,**C**) and HSB-2 cells (**B**,**D**) were pre-activated or not with SDF1α for 1 h and then seeded on VCAM-1, fibronectin or BSA for 4 h. The non-adherent cells were removed by washing three times with the RPMI medium and adherent cells were treated for 48 h with 50 ng/mL and 10 ng/mL of doxorubicin for Jurkat and HSB-2 cells respectively. Apoptosis was determined by annexin V staining and FACS analysis. Results represent mean values ± S.D. of three independent experiments. ** *p* < 0.01 and *** *p* < 0.001 determined by the Student’s *t*-test.

To study the role of the VCAM-1-binding integrin VLA-4 and fibronectin-binding integrin VLA-5 in chemoresistance of T-ALL cells, we assessed the apoptotic response to doxorubicin of Jurkat and HSB-2 cells adhering on VCAM-1 and fibronectin. The results show that attachment to VCAM-1 reduces doxorubicin-induced apoptosis of Jurkat and HSB-2 cells by 32% and 21% respectively, suggesting that VCAM-1 induces resistance of T-ALL cells to doxorubicin (Figure 3A,B). In contrast, cell adhesion to fibronectin had no effect in either cell line (Figure 3C,D), which is in agreement with our previous study [25].

We also evaluated the impact of SDF1α on T-ALL chemoresistance. We found that SDF1α alone had no effect on the response of T-ALL cells to doxorubicin (Figure 3). In addition, it did not improve the chemoresistance induced by VCAM-1 in Jurkat cells and HSB-2 cells (Figure 3A,B). Although SDF1α increases the adhesion of T-ALL cell lines to fibronectin, it did not induce their chemoresistance (Figure 3C,D). Together, these results show that VLA-4 interaction with VCAM-1 but not VLA-5 interaction with fibronectin promotes T-ALL cell chemoresistance. 

**Figure 4:**
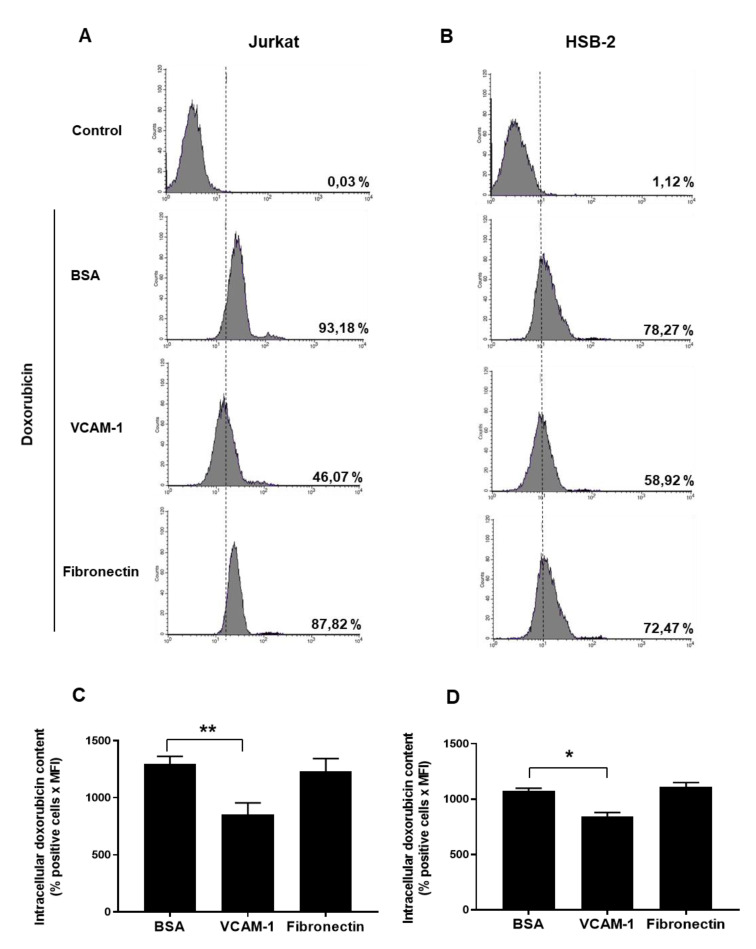
VCAM-1 but not fibronectin enhances doxorubicin efflux in human T-ALL cell lines. Jurkat and HSB-2 cells were seeded on VCAM-1, fibronectin or BSA for 4 h. The non-adherent cells were removed by washing with RPMI medium and the remaining cells were treated for 2 h with 100 ng/mL of doxorubicin. The cells were then washed and intracellular doxorubicin content was analyzed by FACS using the FL-2 channel. (**A**,**B**) Representative FACS profiles of intracellular doxorubicin content in Jurkat and HSB-2 cells. (**C**,**D**) Quantification of intracellular doxorubicin content in Jurkat and HSB-2 cells was determined by the percentage of doxorubicin-positive cells x MFI. Results represent mean values ± S.D. of three independent experiments. * *p* < 0.05 and ** *p* < 0.01 were determined by the Student’s t-test.

Having determined the role of VCAM-1 in T-ALL cell resistance to doxorubicin, we studied the mechanism of chemoresistance induced by VCAM-1. Activation of drug efflux via membrane transporters of the ABC transporter family is a major mechanism of drug resistance [29,30,31]. To determine if T-ALL cells attached to VCAM-1 activate this mechanism, we assessed the intracellular doxorubicin content in these cells by flow cytometry. The results show that VCAM-1 reduces intracellular doxorubicin content in Jurkat and HSB-2 cells by 38% and 22% on average respectively suggesting that VCAM-1 induces chemoresistance by promoting doxorubicin efflux (Figure 4). However, fibronectin had no effect consistent with its inability to protect the cells from doxorubicin-induced apoptosis. 

### 3.3. PYK2 but Not FAK Is Involved in VCAM-1-Induced Doxorubicin Resistance

**Figure 5:**
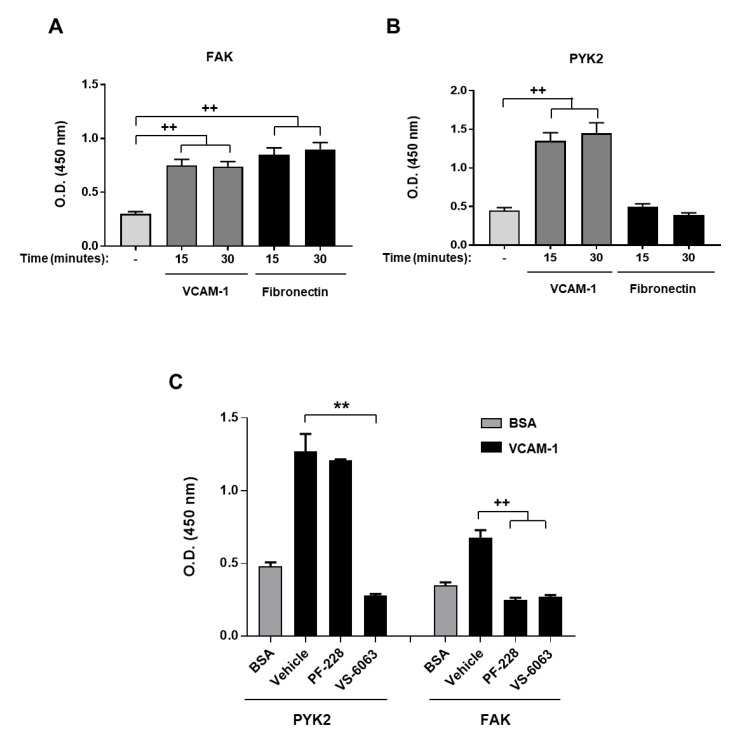
Differential activation of FAK and PYK2 in T-ALL cells. Jurkat cells were seeded on VCAM-1 or fibronectin for 15 and 30 min or on BSA (−) for 30 min. The cells were lysed and phosphorylation of FAK (**A**) and PYK2 (**B**) was determined by specific ELISA assays. (**C**) The cells were incubated with the vehicle (−) or with 0.5 µM of the FAK inhibitor PF-228 and the dual FAK/PYK2 inhibitor VS-6063 for 1 h and then seeded on VCAM-1 for 30 min. FAK and PYK2 activations were then determined by ELISA assays. Results represent mean values ± S.D. of three independent experiments. ** *p* < 0.01 determined by the Student’s *t*-test. **^++^** *p* < 0.01 determined by the two-way ANOVA test.

To further understand how VLA-4 induces chemoresistance in T-ALL cells, we examined the implication of focal adhesion kinases that are known to play a central role in integrin signaling. We therefore studied the activation of both FAK and PYK2 and used the FAK inhibitor PF-228 and the dual FAK/PYK2 inhibitor VS-6063 to determine which kinase is implicated in VCAM-1-induced chemoresistance. Jurkat cell adhesion to VCAM-1 induces the activation of both FAK and PYK2 (Figure 5A,B). However, cell adhesion to fibronectin led only to FAK activation (Figure 5A,B).

Extending the time of stimulation with fibronectin did not affect PYK2 activation. Inhibitory experiments indicate that VCAM-1-induced FAK activation is abrogated by both the FAK (PF-228) and the FAK/PYK2 (VS-6063) inhibitors, whereas PYK2 activation is abrogated only by the VS-6063 inhibitor (Figure 5C).

**Figure 6:**
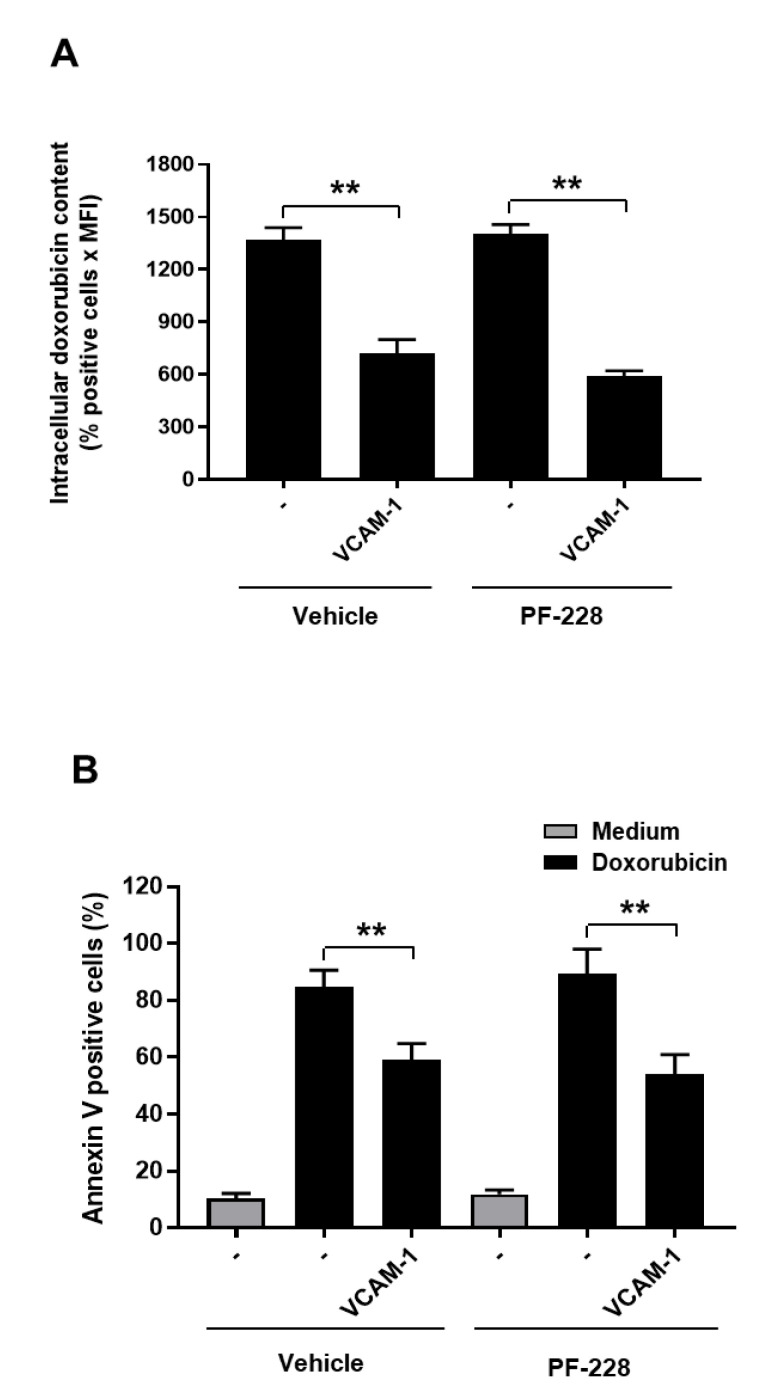
T-ALL chemoresistance mediated by VCAM-1 is FAK-independent. (**A**) The FAK inhibitor did not affect VCAM-1-induced drug efflux. Jurkat cells were treated with vehicle (−) and the FAK inhibitor PF-228 (0.5 µM) for 1 h and then seeded on VCAM-1 for 4 h. Adherent cells were treated for 2 h with 100 ng/mL of doxorubicin and intracellular doxorubicin content was determined by FACS analysis. (**B**) The FAK inhibitor did not affect VCAM-1-induced T-ALL chemoresistance. The cells were treated as in (**A**) and apoptosis was determined after 48 h of treatment with 50 ng/mL of doxorubicin by annexin V staining and FACS analysis. Results represent mean values ± S.D. of three independent experiments. ** *p* < 0.01 determined by the Student’s *t*-test.

We then used the specific FAK inhibitor PF-228 to determine if FAK intervenes in chemoresistance and doxorubicin efflux induced by VCAM-1. The analysis of intracellular doxorubicin content shows that PF-228 had no effect on the doxorubicin efflux induced by VCAM-1 as the inhibitor did not increase the intracellular doxorubicin content in Jurkat T cells adhered on VCAM-1 (Figure 6A). Moreover, the PF-228 has also no effect on chemoresistance induced by VCAM-1 since it does not sensitize the cells adhered on VCAM-1 to doxorubicin-induced apoptosis (Figure 6B).

**Figure 7:**
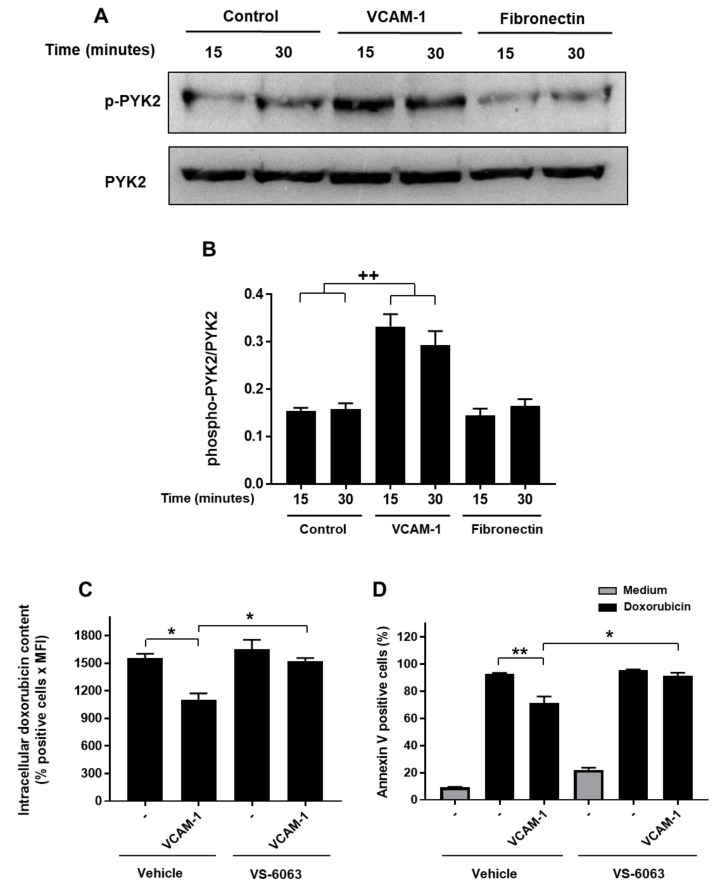
VCAM-1 induces doxorubicin resistance in a PYK2-dependant manner. (**A**) VCAM-1 but not fibronectin induces PYK2 phosphorylation. Jurkat cells were seeded on VCAM-1 or fibronectin for 15 min and 30 min or on BSA (−) for 15 min. The cells were then lysed and cell lysates were analyzed by Western blot with anti-phosphorylated PYK2 (Tyr-402) antibody. The immunoblots were stripped and re-probed with total anti-PYK2 antibody to ensure equal loading. Representative immunoblot from three independent experiments. (**B**) Densitometric quantification of PYK2 activation was determined by the ratios between the levels of phosphorylated-PYK2 and total PYK2 (n = 3). (**C**) PYK2 inhibition blocks doxorubicin efflux induced by VCAM-1. Jurkat cells were treated with vehicle or with the PYK2 inhibitor VS-6063 (0.5 µM) for 1 h and then seeded on VCAM-1 for 4 h. The adhered cells were then treated for 2 h with 100 ng/mL of doxorubicin and intracellular doxorubicin content was determined by FACS analysis. (**D**) PYK2 inhibitor abrogates the protective effect of VCAM-1. The cells were treated as in (**C**) and apoptosis was determined after 48 h of treatment with 50 ng/mL of doxorubicin by annexin V staining and FACS analysis. Results represent mean values ± S.D. of three independent experiments. * *p* < 0.05 and ** *p* < 0.01 were determined by the Student’s *t*-test. **^++^** *p* < 0.01 determined by the two-way ANOVA test.

We then focused on the regulation of the FAK-related kinase PYK2 and confirmed its differential activation by VCAM-1 and fibronectin by Western blot analysis. The results show that VCAM-1 but not fibronectin induces PYK2 phosphorylation after 15 min and 30 min of stimulation (Figure 7A). The densitometry quantification shows that the ratio between phosphorylated-PYK2 and total PYK2 increases by two-fold in the presence of VCAM-1 but not fibronectin (Figure 7B). 

To examine whether PYK2 is involved in chemoresistance induced by VCAM-1, we studied the effect of VS-6063, a dual PYK2/FAK inhibitor. VCAM-1-induced doxorubicin efflux is blocked in the presence of VS-6063 as the doxorubicin content in cells adhered on VCAM-1 and treated with VS-6063 is similar to that of cells treated only with doxorubicin (Figure 7C). 

In order to determine if the effect of VS-6063 on doxorubicin efflux translated into the regulation of chemoresistance, we analyzed the effect of VS-6063 on doxorubicin-induced apoptosis in Jurkat cells adhered on VCAM-1. We found that treatment of T-ALL cells with VS-6063 abrogates chemoresistance induced by VCAM-1. Indeed, the apoptosis rate of cells adhered on VCAM-1 and treated by VS-6063 is similar to that of cells treated only with doxorubicin (Figure 7D). Interestingly, there was no significant effect of VS-6063 alone on intracellular doxorubicin content or on Jurkat cells apoptosis. Taken together, these results indicate that VCAM-1 induces doxorubicin efflux and chemoresistance of T-ALL cells via PYK2. 

## 4. Discussion

VLA-4 is an important cell adhesion molecule in the immune system and has recently been implicated in the chemoresistance of hematological malignancies including B cell neoplasms and myeloid leukemia [15,18,32]. However, the role of VLA-4 in the survival and chemoresistance of T-ALL has been investigated less. In this study, we report that VLA-4 interaction with VCAM-1 induces doxorubicin resistance of T-ALL cells, whereas adhesion to fibronectin had no effect. We further found that this is due to VLA-4-mediated doxorubicin efflux, which is dependent on PYK2 but not FAK. The prosurvival effect of VLA-4 is more pronounced in the mature T-ALL cell line Jurkat than in the immature HSB-2 T-ALL cell line. This could be due to the better capacity of Jurkat cells to attach to VCAM-1, suggesting that VCAM-1-induced chemoresistance could be more relevant for T-ALL patients displaying high levels of α4 and greater abilities to attach to VCAM-1. 

We have previously reported that the adhesion of T-ALL cells to fibronectin did not regulate their sensitivity to drug-induced apoptosis [25]. Herein, we further found that despite SDF1α increasing T-ALL cell adhesion to fibronectin, it did not induce chemoresistance of T-ALL cells adhering to fibronectin. Similarly, SDF1α also had no effect on VCAM-1-induced T-ALL chemoresistance, suggesting that the SDF1α chemotactic and VCAM-1/fibronectin adhesion signaling pathways are not coupled in T-ALL chemoresistance. 

However, cross-linking of VLA-4 and VLA-5 with recombinant fibronectin ligands has been shown to equally induce doxorubicin resistance in Jurkat T cells [33]. The fact that the whole fibronectin molecule has no effect on chemoresistance suggests that fibronectin could have engaged additional receptors, which might block the protective effect of VLA-5. It is also likely that fibronectin does not crosslink VLA-5 on T-ALL cells as does the recombinant Fn9.11 fragment, resulting in differential signaling capacities. Although additional studies are warranted to determine the exact contribution of VLA-5 to T-ALL chemoresistance, our results firmly show that VLA-4 interaction with its natural ligand VCAM-1 also has a role in the chemoresistance of the T-ALL malignancy.

We then examined the mechanisms by which VLA-4 induces chemoresistance. Drug efflux mediated by ABC transporters is a major mechanism in cancer drug resistance [29,30,34]. We found that in both T-ALL cell lines, attachment to VCAM-1 induces doxorubicin efflux. We have not determined which ABC transporter is activated by VLA-4 but we previously found that β1 integrin interaction with collagen or matrigel induces T-ALL chemoresistance by activating ABCC1 (ATP Binding Cassette Subfamily C Member 1) [26,35]. In support, VCAM-1-induced doxorubicin efflux is reversed by the ABCC1 inhibitor MK-571 (Supplementary Figure S2). Thus, we propose that ABCC1, which is the most expressed ABC transporter in T-ALL cells including Jurkat and HSB-2 [35], could also be the target of VLA-4. Along these lines, recent studies in acute myeloid leukemia and in breast cancer reported that β1 integrins are also activators of drug efflux and ABC transporters [36,37,38], suggesting a general mechanism. Thus, one major mechanism by which cell adhesion-mediated drug resistance proceeds is via the β1-integrin/ABC transporter pathway.

We then determined the signaling pathways activated by VLA-4 in T-ALL chemoresistance. Focal adhesion kinases play a central role in cell adhesion mediated signaling and in cancer. We therefore considered the implication of FAK and its related member PYK2. Although T-ALL cell adhesion to VCAM-1 and fibronectin activated FAK, inhibition of FAK did not abrogate the effect of VLA-4 on T-ALL cell chemoresistance and doxorubicin efflux indicating the non-implication of FAK. In contrast to T-ALL cells, FAK has been associated with chemo- and radio-resistance in solid tumors suggesting a cell-type specific function in chemoresistance [39,40,41,42]. 

We found that PYK2 inhibition abolished the ability of VLA-4/VCAM-1 interaction to induce doxorubicin efflux and resistance indicating that PYK2 plays a major role in VLA-4-induced T-ALL chemoresistance. In contrast to VCAM-1, attachment of T-ALL cells to fibronectin did not activate PYK2, which is in line with the failure of fibronectin to induce doxorubicin efflux and resistance. Thus, differential PYK2 activation could be one mechanism contributing to explain the differential role of VLA-4 and VLA-5 in T-ALL chemoresistance. Previous studies reported that fibronectin is capable of activating PYK2 in FAK^-^/_-_ fibroblasts and in epithelial cells [43]. This could be explained by the different cell types considered and the expressed integrin profile and connection to intracellular pathways, which could be different.

In agreement with our findings in T-ALL cells, it has been shown that PYK2 mediates chemoresistance of ovarian cancer cells induced by IL-6 [44] and PYK2 has been implicated in myeloma survival and progression [45,46]. Together, these studies indicate that PY2K is a critical pathway in microenvironment-induced cancer cell survival and chemoresistance.

PYK2 plays an important role in cell adhesion, which therefore could explain its role in VCAM-1-induced chemoresistance. However, it is not excluded that it can also directly activate doxorubicin efflux and lead to chemoresistance. Although T-ALL cells attach well to fibronectin, they were not protected from doxorubicin. Therefore, inducing cell adhesion in general is not sufficient to induce doxorubicin efflux and chemoresistance in T-ALL cells.

In summary, our study showed that VLA-4 could be an important adhesion molecule in T-ALL chemoresistance. The fact that VLA-2-mediated adhesion to collagen also promoted T-ALL cell chemoresistance [25], suggests a redundancy between β1 integrin family members in promoting T-ALL cell survival and chemoresistance. In addition, PYK2-induced drug efflux is also observed in β1 integrin binding to extracellular matrix and induction of T-ALL chemoresistance [26]. This suggests that targeting PYK2 could be more efficient to overcome T-ALL chemoresistance than targeting each integrin separately.

## 5. Conclusions

Our study showed the role of VLA-4 interaction with VCAM-1 in T-ALL chemoresistance. We provided a novel mechanism by which VLA-4 contributes to cancer chemoresistance, which is through induction of PYK2-dependent drug efflux. However, T-ALL cell adhesion to fibronectin, which is dependent on VLA-5, did not activate PYK2 and had no effect on doxorubicin efflux and chemoresistance. Therefore, our study suggests that targeting the VLA-4/PYK2 pathway may represent a novel therapeutic target in T-ALL.

## Data Availability

Not applicable.

## Supplementary Materials

The following are available online at https://www.mdpi.com/article/10.3390/cancers13143512/s1, Supplementary Figure S1: VLA-5 (α5 integrin) but not VLA-4 (α4 integrin) mediates adhesion of TALL cells to fibronectin, Supplementary Figure S2: ABCC1 blockade inhibits VCAM-1-induced doxorubicin efflux, Supplementary Figure S3: Uncropped western blots for Figure 7.
