# Supplementary material for: VLA-4 Induces Chemoresistance of T Cell Acute Lymphoblastic Leukemia Cells via PYK2-Mediated Drug Efflux
Source: Cancers (Basel). 2021 Jul 14;13(14):3512. doi: 10.3390/cancers13143512 (PMC8307050; doi:10.3390/cancers13143512)

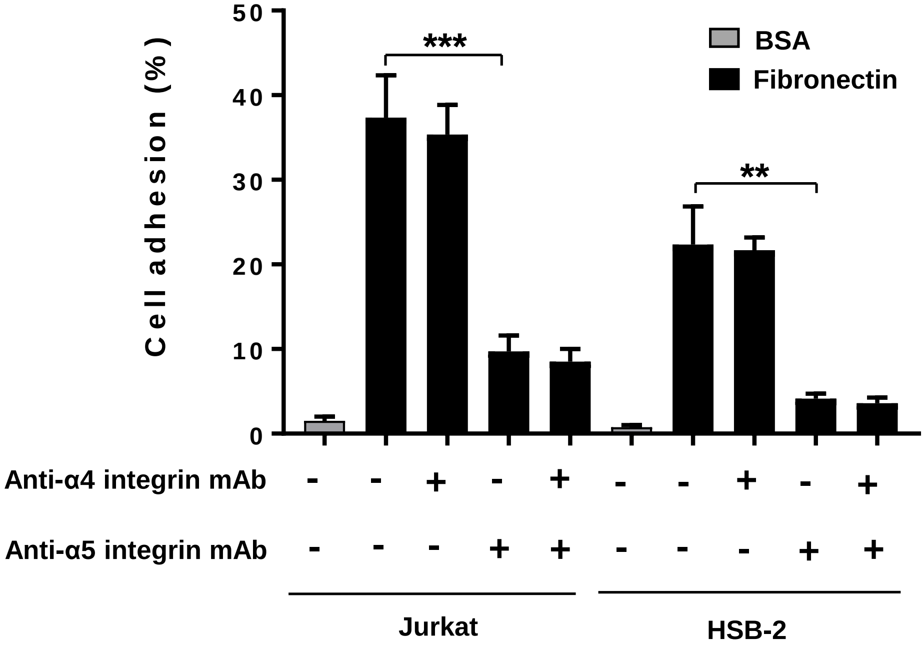

Supplementary figure S1: VLA-5 (α5 integrin) but not VLA-4 (α4 integrin) mediates adhesion of T-ALL cells to fibronectin. Jurkat and HSB-2 cells were pre-treated for 1h with 10 µg/ml of blocking antibodies against α4 integrin (P1H4) and α5 integrin (P1D6) as indicated and then cultured for 4h in wells coated with fibronectin or BSA. The cells were then washed three times and the remaining cells were counted. Results represent mean values percentages of adherent cells ± S.D. of three independent experiments. **P<0.01 and ***P<0.001 (Student's t-test).

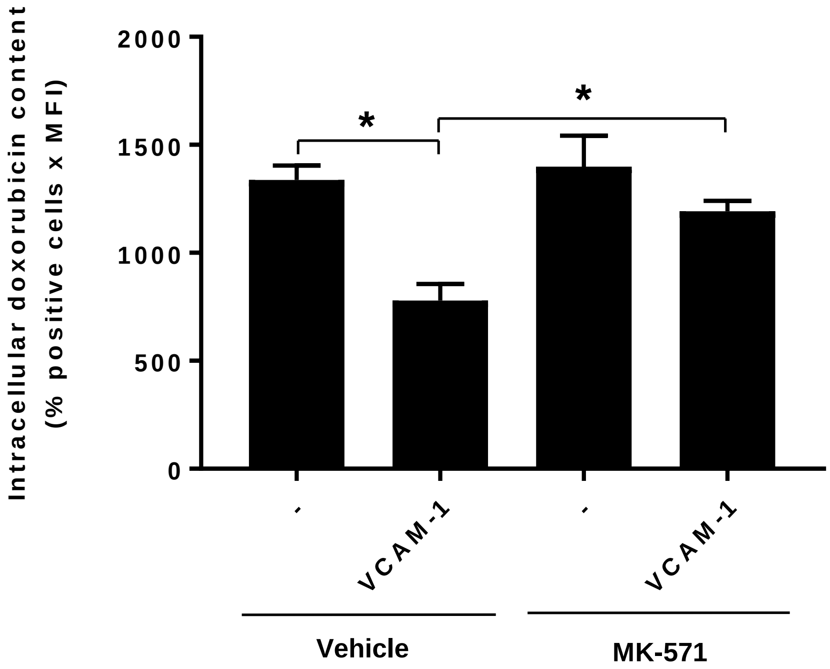

Supplementary figure S2: ABCC1 blockade inhibits VCAM-1-induced doxorubicin efflux. Jurkat cells were pre-treated for 1h with ABCC1 inhibitor MK-571 (10µM) or vehicle. The cells were then seeded on VCAM-1 or BSA(-) for 4h. The non-adherent cells were removed by washing with RPMI medium and the remaining cells were treated for 2h with 100 ng/ml of doxorubicin. The cells were then washed and intracellular doxorubicin content was analyzed by FACS using the FL-2 channel. Quantification of intracellular doxorubicin content was determined by the percentage of doxorubicin-positive cells x MFI. Results represent mean values ± S.D. of three independent experiments. *$P<0.05$ (Student's t-test).

Supplementary figure S3: Uncropped western blots for figure 7

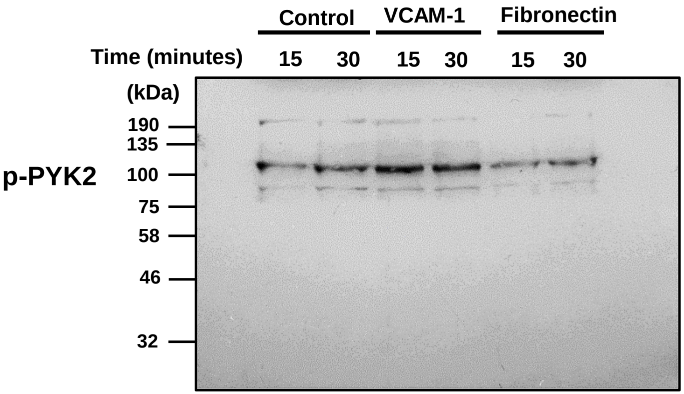

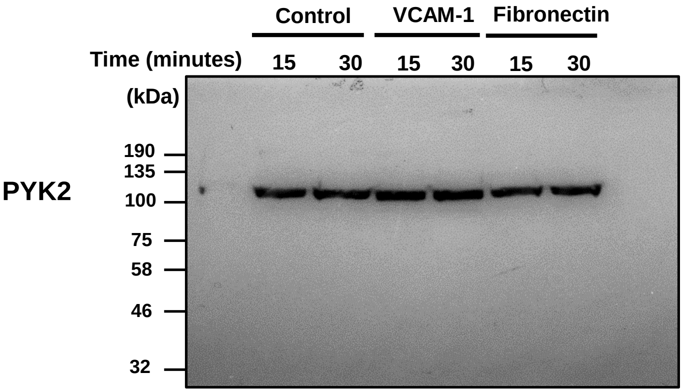

Supplement: Supplementary file 1 [file cancers-13-03512-s001.zip › cancers-1233118-supplementary.pdf]
